# The Association Between Brain Volumes and Posttraumatic Stress Disorder in Intensive Care Unit Survivors: A Preliminary Study

**DOI:** 10.3389/fnins.2020.00690

**Published:** 2020-06-30

**Authors:** Kristina Stepanovic, Baxter Rogers, Amy L. Kiehl, E. Wesley Ely, James Jackson, Jo Ellen Wilson, for the VISIONS Investigators and VISualizing Icu SurvivOrs Neuroradiological Sequelae Cohort Study

**Affiliations:** ^1^Center for Critical Illness Brain Dysfunction and Survivorship, Vanderbilt University Medical Center, Nashville, TN, United States; ^2^Department of Medicine, Vanderbilt University Medical Center, Nashville, TN, United States; ^3^Vanderbilt University Institute of Imaging Science, Vanderbilt University School of Medicine, Nashville, TN, United States; ^4^Department of Radiology and Radiological Sciences, Vanderbilt University Medical Center, Nashville, TN, United States; ^5^Department of Biomedical Engineering, Vanderbilt University, Nashville, TN, United States; ^6^Department of Psychiatry and Behavioral Sciences, Vanderbilt University Medical Center, Nashville, TN, United States; ^7^Department of Medicine, Division of Pulmonary and Critical Care, and the Center for Health Services Research, Vanderbilt University Medical Center, Nashville, TN, United States; ^8^Veteran’s Affairs TN Valley, Geriatrics Research, Education and Clinical Center, Nashville, TN, United States

**Keywords:** posttrauamtci stress disorder, brain volume, critical illness, amygdala, hippocampus

## Abstract

**Introduction:**

Millions of Americans are admitted to the intensive care unit (ICU) per year. Many survivors of the ICU will experience posttraumatic stress disorder (PTSD); although volumetric hippocampal and amygdala studies have been conducted in other trauma survivors (i.e., veterans), the association between PTSD symptoms and hippocampal and amygdala volumes in ICU survivors has not been described. We hypothesize that the severity of posttraumatic stress symptoms in ICU survivors is associated with lower volumes of both the hippocampus and amygdala at 3 and 12 months.

**Methods:**

Secondary analysis of the VISIONS study, a prospective sub-study of the BRAIN-ICU cohort, which included survivors of critical illness. The PTSD Checklist Specific was used at 3 and 12 months to evaluate the ICU as a traumatic experience. A Philips Achieva 3T MRI scanner was used to scan patients at both discharge and 3 months. To compare median brain volumes at discharge and 3 months for those with and without PTSD symptomatology, we used a Kruskal–Wallis (KW) test.

**Results:**

At 3 month follow up, three patients had PTSD symptomatology and *N* = 1 at 12 month follow up. There was no difference between median brain volumes (hippocampus or amygdala) between individuals with PTSD symptomatology at either 3 or 12 months (*p*-values > 0.05).

**Discussion:**

Although our study did not reveal significant differences in brain volumes between PTSD patients and non-PTSD patients, sample size was a major limitation and larger scale studies should be undertaken to elucidate possible neurobiological markers of PTSD in ICU survivors.

## Introduction

Annually, nearly 800,000 individuals are admitted to an intensive care unit (ICU) on mechanical ventilation at the cost of over 27 billion dollars (Halpern and Pastores, 2015). Of those who survive, many will go on to experience posttraumatic stress disorder (PTSD; Parker et al., 2015;Patel et al., 2016). Posttraumatic stress disorder is a mental health condition that may develop in individuals who experience or witness a traumatic, life-threatening event and is characterized by re-experiencing, hyperarousal, avoidance, memory impairment, flashbacks, mood labiality, and hypervigilance (APA, 2013). Although critical illness treatments are aimed at sustaining life, they can be stressful, and can include a range of terrifying experiences such as respiratory insufficiency, loss of ability to communicate, and may include altered cognition, hallucinations and delusions (Jackson et al., 2014). Potential risk factors for the development of PTSD after the ICU include: pre-morbid psychiatric history, sedation, mechanical ventilation, physical restraints, delirium, delusional memories, loss of control, and agitation (Wade et al., 2013).

To our knowledge, no neuroimaging studies have explored the association between post-traumatic stress and neurological changes *in survivors of the ICU*, although this has been well-studied in other populations, which have implicated several key brain regions in the development of the disorder including the hippocampus, amygdala, and the prefrontal cortex (Karl et al., 2006; Woon et al., 2010; O’Doherty et al., 2015). Woon et al.’s (2010) metanalysis of 39 volumetric studies found lower hippocampal volumes in both subjects with PTSD and trauma-exposed subjects without a PTSD diagnosis. In a large study of veterans, subjects with PTSD had a smaller volume for both the right and left amygdala (Morey et al., 2012).

By understanding more about PTSD pathophysiology, further steps can be taken to attempt to prevent or treat PTSD (Long et al., 2014). The purpose of this investigation is to explore the severity of posttraumatic stress symptoms in association with hippocampal and amygdala volumes in survivors of the ICU. We hypothesize that the severity of posttraumatic stress symptoms in ICU survivors is associated with lower volumes of both the hippocampus and amygdala.

## Methods

We undertook a secondary analysis of the VISIONS (VISualizing Icu SurvivOrs Neuroradiological Sequelae) study (Gunther et al., 2012), a prospective convenience sample sub-study of the BRAIN-ICU cohort (Pandharipande et al., 2013). The IRB at Saint Thomas Hospital and Vanderbilt University approved the study protocol. Enrolled patients had survived ICU treatment with mechanical ventilation or vasopressors in the context of severe critical illness and were agreeable to participate in this neuroimaging sub-study.

To be eligible to participate in the study, adult patients had to meet the following criteria: surviving in the ICU in cardiac, surgical, or medical ICU at Saint Thomas Hospital or Vanderbilt University Hospital, both located in Nashville, TN. The exclusion criteria were as follows: deafness, blindness, delirium at hospital discharge, dementia, anoxic brain injury, TBI, known brain lesions, MRI contraindications, claustrophobia, and weight of more than 300 pounds.

Patients were screened for preexisting PTSD before they left the hospital. The PTSD Checklist Specific (PCL-S; Blanchard et al., 1996) was used to identify a traumatic event and categorized severity based on their score. The PCL-S was used at 3 and 12 months after discharge to evaluate the ICU as a traumatic experience. Our PTSD threshold was a PCL-S score of greater than or equal to 30, as we wanted to capture all possible patients with significant symptoms of PTSD (Magruder et al., 2015).

A Philips Achieva 3T MRI scanner was used to scan patients at both discharge and 3 month follow-up. Scanning included T1-weighted 3D turbo field echo image covering the whole brain, 170 slices, TR = 8.0 ms, TE = 3.7 ms, SENSE factor = 2, voxel size = 1 mm isotropic, FOV = 256 × 256 × 170. Study staff computed the regional volumes with an established software pipeline, without adjusting any parameter settings, in particular, without adjusting settings after reviewing the results. A multi-atlas segmentation algorithm (Asman and Landman, 2013) using a set of 45 manually labeled atlases (Neuromorphometrics, Inc., Somerville, MA, United States) was applied to parcellate each person’s T1-weighted structural image. The regions extracted followed brainCOLOR and included hippocampus, amygdala, and total intracranial volume. The hippocampus and amygdala regions were further refined using a multi-scale approach based on an additional set of atlases (Plassard et al., 2017). The data was also visually inspected to ensure accuracy.

Study data were collected and managed using REDCap electronic data capture tools hosted at Vanderbilt University (Harris et al., 2009, 2019). Amygdala and hippocampal volumes were defined as right plus left hemispheres for each respective structure. To compare median brain volumes at discharge and 3 months for those with and without significant PTSD symptomatology (PCL-S = 30) at 3 and 12 months, we used a Kruskal–Wallis (KW) equality-of-populations rank test.

## Results

The median age for our sample was 58.5 (52.6, 63.7) (Table 1). One-third of the sample was female, and 90% were Caucasian. Fifty-seven percent of individuals (*N* = 12) had at least 1 prior mental health diagnosis, with *N* = 2 having a prior history of PTSD. One-third of individuals experienced delirium during their critical illness. At 3 month follow up, there were three patients with PTSD symptomatology and *N* = 1 at 12 month follow up. Hippocampal and amygdala volumes at discharge and 3 month follow-up are reported in Table 1. There was no difference between median brain volumes (hippocampus or amygdala) between individuals with PTSD symptomatology at either 3 or 12 months (*p*-values for all tests > 0.05).

**TABLE 1:** Patient demographic and clinical characteristics.

**Variable**	*N* (%) or median (IQR)*
	*N* = 21**
**Age (years)**	58.5 (52.6, 63.7)
**Gender**	
Female	7 (33%)
Male	14 (67%)
**Race**	
Black/African American	2 (10%)
White/Caucasian	19 (90%)
**ICU type**	
Medical	10 (48%)
Surgical	11 (52%)
**Charlson comorbidity index**	1 (1, 3)
**SOFA score (at enrollment)**	10 (8, 12)
**Diagnosis at admission**	
Sepsis/ARDS due to infection or septic shock	7 (33%)
ARDS without infection	2 (10%)
CHF/Cardiogenic shock	1 (5%)
Hepatobiliary/pancreatic surgery	6 (29%)
COPD/Asthma	3 (14%)
Vascular surgery	2 (10%)
**Delirium**	
Number of patients with delirium	13 (62%)
Length of delirium (in days, for those affected)	1 (1, 5)
**Preexisting mental health diagnosis**	
PTSD	2 (10%)
Alcohol use disorder	3 (14%)
Depression	10 (48%)
Bipolar disorder	1 (5%)
Personality disorder	1 (5%)
**ICU length of stay (days)**	4 (1, 5)
**Hospital length of stay (days)**	9 (6, 12)
**PTSD (yes, score = 30)**	
3 month follow up (*N* = 21)	3 (14%)
12 month follow up (*N* = 13)	1 (8%)
**PCL total score**	
3 month follow up	19 (18, 24)
12 month follow up	20 (17, 28)
**Amygdala volume (total volume; mm^3^)**	
discharge	2110 (2004, 2205)
3 month follow up	2079 (1921, 2192)
**Hippocampus volume (total volume; mm^3^)**	
discharge	5779 (5359, 6101)
3 month follow up	5674 (5182, 6240)

*IQR, Interquartile range. **N = 21 is the group of patients who had both a MRI at discharge and a 3 month PCL-S complete. Only *N* = 13 had both a 3 month MRI and 12 month PCL. Scores on the Sequential Organ Failure Assessment (SOFA) range from 0 to 24 (from 0 to 4 for each of six organ systems), with higher scores indicating more severe organ dysfunction. We used a modified SOFA score, which excluded the Glasgow Coma Scale components, since coma was included separately in our models. Scores on the Charlson comorbidity index range from 0 to 33, with higher scores indicating a greater burden of illness; a score of 1 or 2 is associated with mortality of approximately 25% at 10 year. ARDS, Acute Respiratory Distress Syndrome; CHF, Congestive Heart Failure; COPD, Chronic Obstructive Pulmonary Disease.

## Discussion

Millions of Americans are admitted to the ICU per year – nearly 800,000 of which are on mechanical ventilation (Halpern and Pastores, 2015). Many survivors will develop PTSD, and although volumetric neurological studies have been conducted in veterans and other trauma survivors, the association between PTSD symptoms and hippocampal and amygdala volumes in ICU survivors has not yet been studied before this secondary analysis. We hypothesized that the severity of posttraumatic stress symptoms in ICU survivors is associated with lower volumes of both the hippocampus and amygdala at 3 and 12 months. Using a secondary analysis of the VISIONS study, we found at 3 month follow up, three patients had PTSD symptomatology and *N* = 1 at 12 month follow up. We did not find a significant difference between median brain volumes (hippocampus or amygdala) between individuals with PTSD symptomatology at either 3 or 12 months.

Although our study did not reveal significant differences in brain volumes between PTSD patients and non-PTSD patients, sample size was a major limitation and larger scale studies should be undertaken to elucidate possible neurobiological markers of PTSD in ICU survivors. Large metanalyses and studies in veterans have revealed trauma exposure and PTSD can lead to lower volumes in both the hippocampus and amygdala (Woon et al., 2010; Morey et al., 2012). Additionally, by using a less conservative threshold for PTSD on the PCL, we cannot say for certain that patients in our sample had PTSD. With a larger scale study, more conservative cut points (e.g., >50) could be employed. Additionally, we do not have imaging pre-critical illness; thus, we cannot know whether brain volumes were directly related to their critical illness.

Further studies should investigate the role of sedation in the evolution of delirium and PTSD. More research is needed to investigate the neurological changes in ICU survivors with PTSD, to understand the pathophysiology of the disease, inform clinical treatment of survivors, and aid in understanding risk factors (Patel et al., 2016).

## Data Availability Statement

The datasets generated for this study are available on request to the corresponding author.

## Ethics Statement

The studies involving human participants were reviewed and approved by the Institutional Review Boards at Vanderbilt University Medical Center and St. Thomas Hospital. Written informed consent was provided by all study participants or their independent examiner where specified by the IRB.

## Author Contributions

All authors listed above have contributed substantially to the conception or design of the work; or the acquisition, analysis, or interpretation of data for the work and have participated in drafting the work or revising it critically for important intellectual content. Additionally, each author has given their approval to the final version of the manuscript and has agreed to be accountable for all aspects of the work in ensuring that questions related to the accuracy or integrity of any part of the work are appropriately investigated and resolved.

## Conflict of Interest

JW would like to acknowledge salary support from the Vanderbilt Faculty Research Scholars Program (1KL2TR002245), HL111111 and GM120484. EE and JJ as well as AK all receive funding for their time working on this investigation from AG035117 and HL111111. EE would additionally like to acknowledge salary support from the Tennessee Valley Healthcare System Geriatric Research Education and Clinical Center (GRECC). EE will also disclose additional funding for his time from AG027472 and having received honoraria from Orion and Hospira for CME activity; he does not hold stock or consultant relationships with those companies. The authors would like to acknowledge the following: this work was conducted in part using the resources of the Center for Computational Imaging at Vanderbilt University Institute of Imaging Science and the Advanced Computing Center for Research and Education at Vanderbilt University, Nashville, TN, and study data were collected and managed using REDCap electronic data capture tools hosted at Vanderbilt University. The remaining authors declare that the research was conducted in the absence of any commercial or financial relationships that could be construed as a potential conflict of interest.
